# Multifunctional nanocomposite hollow fiber membranes by solvent transfer induced phase separation

**DOI:** 10.1038/s41467-017-01409-3

**Published:** 2017-11-01

**Authors:** Martin F. Haase, Harim Jeon, Noah Hough, Jong Hak Kim, Kathleen J. Stebe, Daeyeon Lee

**Affiliations:** 1 Rowan University, Henry M. Rowan College of Engineering, Glassboro, NJ 08028 USA; 2 Department of Chemical and Biomolecular Engineering, University of Pennsylvania, Philadelphia, PA 19104 USA; 3 Department of Chemical and Biomolecular Engineering, Yonsei University, Seoul, 03722 South Korea

## Abstract

The decoration of porous membranes with a dense layer of nanoparticles imparts useful functionality and can enhance membrane separation and anti-fouling properties. However, manufacturing of nanoparticle-coated membranes requires multiple steps and tedious processing. Here, we introduce a facile single-step method in which bicontinuous interfacially jammed emulsions are used to form nanoparticle-functionalized hollow fiber membranes. The resulting nanocomposite membranes prepared via solvent transfer-induced phase separation and photopolymerization have exceptionally high nanoparticle loadings (up to 50 wt% silica nanoparticles) and feature densely packed nanoparticles uniformly distributed over the entire membrane surfaces. These structurally well-defined, asymmetric membranes facilitate control over membrane flux and selectivity, enable the formation of stimuli responsive hydrogel nanocomposite membranes, and can be easily modified to introduce antifouling features. This approach forms a foundation for the formation of advanced nanocomposite membranes comprising diverse building blocks with potential applications in water treatment, industrial separations and as catalytic membrane reactors.

## Abstract (web summary)

Coating porous membranes with nanoparticles can enhance their separation and antifouling properties, but methods to do so remain complex. Here, Lee and colleagues use solvent transfer-induced phase separation to prepare nanoparticle-functionalized hollow fiber membranes from bicontinuous interfacially jammed emulsion gels.

## Introduction

Membrane separations are promising alternatives to thermal separations because of their high-energy efficiency, their applicability to mixtures containing thermally sensitive materials, and their scalability^1^. For example, water treatment membranes are currently widely used in large-scale reverse osmosis desalination plants and in small-scale personalized ultrafiltration membrane cartridges. Solar powered small-scale ultrafiltration membrane systems are currently employed in remote areas with limited access to safe drinking water or in times of natural disasters and emergencies. Key considerations in the development of new membranes include cost and scalability, high chemical/thermal stability, high selectivity, and high permeability^2^. Membranes should also be designed to separate multicomponent mixtures, be resistant to fouling, and have excellent mechanical and chemical stability^3^.

Nanocomposite membranes composed of mixtures of nanoparticles and polymers have many of these attributes, making them attractive for a variety of applications. Their hydrophilicity^4^, porosity^5^, charge density^6^, thermal and mechanical stability^7, 8^ can be tailored by locating nanoparticles with specific properties and functionality on the surface or within the matrix of the polymer scaffold. In particular, nanocomposite membranes with surface-located nanoparticles can provide unique features such as antibacterial^9^, photocatalytic^10^, or adsorptive^11^ capabilities. For instance, microporous zeolites^12^ have been shown to enhance membrane fluxes at high selectivity for molecular separations^13^. Silver^14^ or copper^15^ nanoparticles also have been demonstrated to introduce antimicrobial functionalities. The photocatalytic activity of surface located titanium dioxide nanoparticles has been shown to introduce self-cleaning properties^16^.

Despite these advantages, nanoparticle-laden polymer membrane fabrication presents major processing challenges, limiting their widespread utilization. Such membranes are typically fabricated by incorporation of nanoparticles directly into the polymer solutions for membrane formation or by modification of preformed polymer membranes with nanoparticles^17^. The former approach is challenging due to unfavorable interactions between the polymers and nanoparticles that drive nanoparticle aggregation. Thus, nanoparticles, introduced to concentrated polymer solutions used for immersion precipitation, tend to distribute non-uniformly on the membrane surface, compromising the membrane structure and properties^18–21^. The latter approach circumvents this nanoparticle aggregation issue, however, modification of pre-formed membranes typically requires multistep processes that exploit specific nanoparticle-/membrane-binding mechanisms^22^. Furthermore, nanoparticles may only coat the very outermost surface of the membrane^23^. Finally, weakly surface-adhered nanoparticles can detach from the membrane during usage and relatively low nanoparticle loading is typically achieved^4, 24, 25^.

Here, we introduce an approach for the fabrication of nanocomposite membranes with nanoparticles densely and uniformly distributed over the entire surface of the porous membrane. Our approach facilitates previously unattainable nanoparticle loadings and placement on membranes and enables the single step fabrication of nanocomposite membranes composed of highly cross-linked polymers (here polyacrylates) with pronounced chemical resistance. This ability to manufacture a highly cross-linked nanocomposite membrane has unique advantages; such a membrane cannot be easily formed via traditional immersion precipitation approaches without specifically functionalized polymers that allow for post-processing^26^.

The highly cross-linked nanocomposite membranes introduced in this work are produced by the combination of recently discovered bicontinuous interfacially jammed emulsion gels (bijels)^27–30^ and some aspects of the classical membrane fabrication techniques known as non-solvent-induced phase separation (NIPS) or immersion precipitation^31–33^. Recently, we have developed solvent transfer-induced phase separation (STRIPS)^34, 35^ to enable continuous manufacturing of STRIPS bijel fibers. Unlike NIPS, STRIPS does not employ polymers, but rather uses liquid monomers for membrane formation. Asymmetric membranes are obtained by arresting the phase separation of the monomers and water with silica nanoparticles that form dense jammed layers at the oil-water interface; the monomers are then polymerized to prepare porous solid membranes. We investigate the morphology evolution during membrane formation, and show that the size of surface pores can be tuned over the range of micrometers down to below 15 nm. Moreover, we show that pH-responsive hydrogel nanocomposite membranes can also be prepared. Lastly, we demonstrate that the high surface coverage of nanoparticles on the membranes offer a rich platform for surface functionalization, illustrated by the attachment of zwitterionic silanes for bio-fouling-resistant membrane formation.

## Results

### Fabrication of STRIPS bijel-derived hollow fiber membrane

**Fig. 1:**
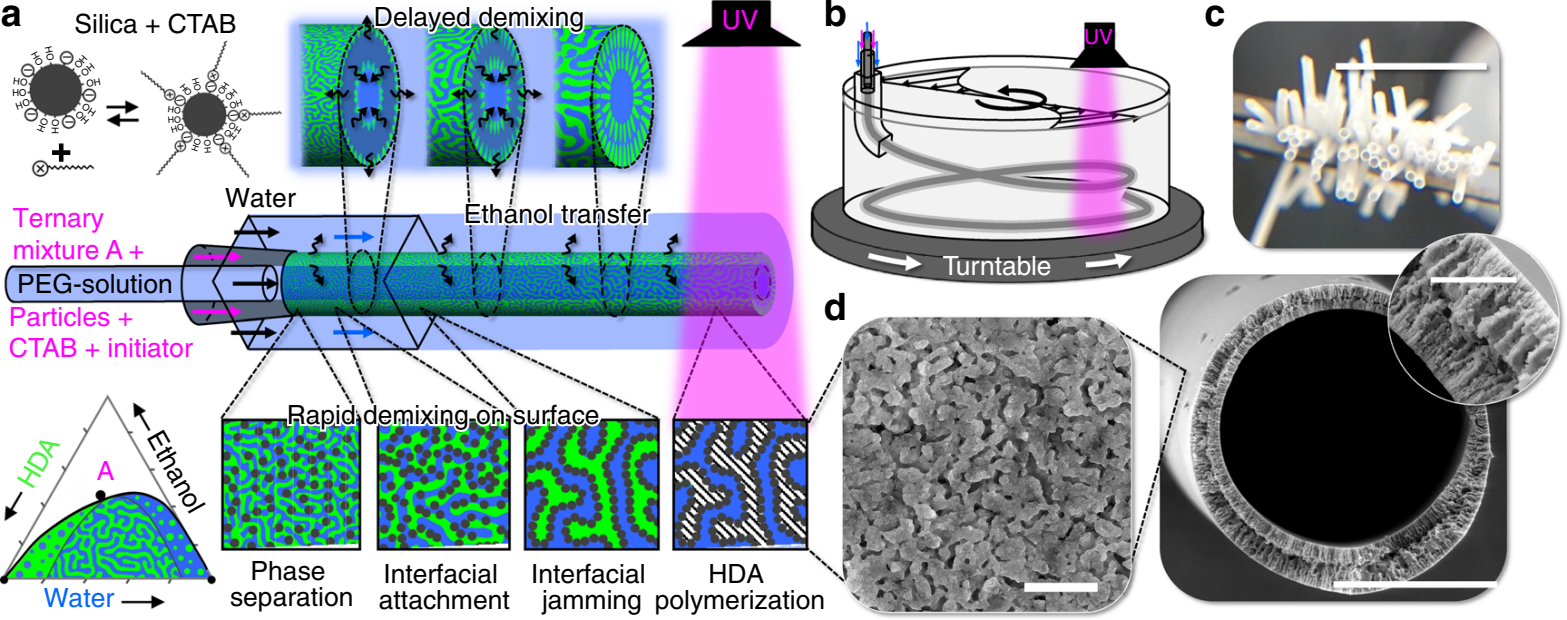
Formation of hollow fiber membranes by STRIPS. **a** Schematics of hollow fiber membrane formation. The co-extrusion of a silica nanoparticle doped monomer solution (ternary fluid) and water (bore fluid and sweeping fluid) yields a hollow fiber. To produce a uniform hollow core, the bore-fluid nozzle must be centered, (as is assured by a square capillary that serves to fix its location, see Supplementary Methods), and the bore fluid viscosity must be adjusted by addition of high molecular mass polyethylene glycol (1 wt% PEG, 600 kDa). Rapid solvent transfer to the outer water phase and to the bore fluid induces phase separation on the fiber surfaces. Delayed solvent transfer in the radial direction generates aligned internal macrovoids. Interfacial nanoparticle jamming yields a nanoparticle-stabilized bicontinuous liquid monomer/water scaffold on the fiber surface. The monomers are polymerized by UV-light irradiation, resulting in a hollow, porous polymer/nanoparticle composite fiber. **b** Aligned fibers are collected in a rotating glass cylinder filled with water. Fiber exiting the orifice is guided to horizontal orientation by the curved outer channel. Prior to polymerization, the hollow nanoparticle-stabilized liquid fibers are fragile (tensile strength ranging from 0.6 to 4 kPa)^35^; flow- and collection bath rotation must be delicately controlled to prevent fracture and defect formation (see Supplementary Methods, Supplementary Note 6). However, upon polymerization, the hollow fiber membrane can be washed and dried without compromising structural integrity. **c** Photograph of dried hollow fiber membrane, scale bar 0.5 cm. **d** SEM micrographs reveal the internal (scale bar 200 µm, inset 20 µm) and surface structures (scale bar 5 µm) of fibers fabricated with ternary flow rate *Q*_T_ = 10 ml h^−l^ and bore fluid flow rate *Q*_B_ = 15 ml h^−1^ at constant water flow rate of 1 ml min^−1^

STRIPS bijel-derived hollow fiber membranes are prepared by STRIPS with a homogeneous mixture of a hydrophobic monomer (hexanediol diacrylate (HDA)), a solvent (ethanol), and water (liquid composition A in ternary diagram Fig. 1a). In this ternary mixture, a surfactant (cetyltrimethyl-ammonium bromide (CTAB)), a photoinitiator (2-hydroxy-2-methylpropiophenone), and colloidally stable nanoparticles (Ludox TMA, silica, diameter 22 nm) are added to enable interfacial jamming and subsequent polymerization of the monomer. The ternary mixture with the surfactant and nanoparticles remains clear, indicating complete mixing among the various components and effective suspension of the nanoparticles (Supplementary Fig. 12). Through a coaxial nozzle, water (bore fluid) and the ternary casting solution are extruded into a co-flowing external stream of water. The bore fluid is introduced to create a uniform, hollow core in the middle of the fiber. The uptake of ethanol into the bore and external water streams depletes the casting solution, and induces phase separation of the HDA and water within the hollow fiber. Adsorption of CTAB on the nanoparticles facilitates their attachment to the interface as well as the formation of a jammed surface structure, resulting in an asymmetric bicontinuous channel network, also known as STRIPS bijel (Fig. 1a)^34, 35^. At a fixed distance from the extrusion nozzle, the hollow STRIPS bijel fiber is converted to a hollow solid membrane by initiating polymerization using ultraviolet (UV) irradiation. We choose HDA as the monomer for our case study in the first part of this paper, but other hydrophobic monomers can be used. HDA provides excellent miscibility in the ternary phase diagram (Fig. 1a) and, upon exposure to UV light, yields a fully cross-linked polymer membrane with high organic solvent resistance.

Structurally, these STRIPS-bijel-derived hollow fiber membranes have some striking similarities to those formed by NIPS^32^. Scanning electron microscopy reveals radially aligned channels that extend from outer and inner surfaces into the membrane (Fig. 1d, inset). This finding clearly illustrates the congeneric nature of STRIPS and NIPS. However, unlike NIPS, the surface structures of STRIPS-bijel-derived membranes are stabilized by the interfacial jamming of nanoparticles rather than by polymer precipitation, which leads to a membrane pore surface that is densely decorated with nanoparticles. As will be shown later, this morphology presents a unique opportunity to impart additional functionality to the membrane.

Intermediate stages of STRIPS bijel formation can be directly visualized by varying the location of UV-initiated polymerization. We place the UV beam at different distances from the extrusion nozzle, thereby capturing different extents of morphology evolution as the membrane forms. The abrupt solidification of the HDA rich-phase fixes the intermediate structure below the surface of the fiber and stops the STRIPS process.

**Fig. 2:**
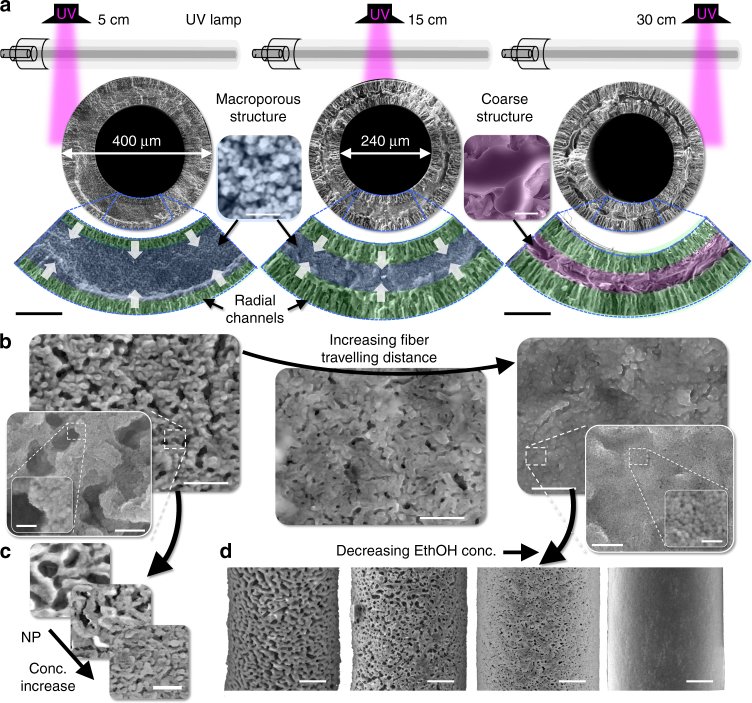
Fiber morphology control. **a** Schematics of UV irradiation to polymerize the membrane structure at different distances from the extrusion nozzle. Below: cross-sectional scanning electron micrographs (SEMs) of the resulting hollow fiber membranes. The magnified sections of the membranes are false-colored in green, blue, and pink. Green highlights regions structured by phase separation. The blue coloration represents a macroporous region with pore sizes in the range of 100–700 nm (Supplementary Note 4). The pink-colored region has a porous structure that is distinct from the green and blue regions. Black scale bars 50 µm. **b** Outer fiber surfaces corresponding to images above. Scale bars 5 µm, inset 1 scale bars 500 nm, inset 2 scale bars 100 nm. Silica mass fraction and CTAB concentration for **a**, **b** are 0.38 g_silica_/g_dry fiber_ and 40 mM, respectively. The mass of silica per mass of dry fiber is calculated based on the initial composition of the extrusion liquid (see Methods section). **c** Fiber surfaces UV-irradiated 5 cm away from the nozzle with different silica nanoparticle mass fractions in the fiber: from top to bottom: 0.26, 0.32, 0.38 g_silica_/g_dry fiber_ under a constant CTAB concentration of 40 mM. Scale bar 5 µm. **d** SEM of fiber surfaces formed at different ethanol concentrations in the continuous water phase (given in vol%) with 0.24 g_silica_/g_dry fiber_ and 33 mM CTAB. Scale bar 20 µm

We find that the radially aligned structures grow from both inner and outer membrane surfaces and coarsen with time (5–30 cm) as shown in Fig. 2a. When polymerization is induced 5 cm away from the nozzle, the radial channels of a solidified hollow fiber membrane extend about 15 µm into the membrane from the surfaces (false colored in green). Between these surface layers, a sponge-like macroporous structure, with pore sizes of 100–600 nm is found (Fig. 2a inset, false colored in blue, see Supplementary Fig. 6). When UV irradiation is performed further downstream, the macroporous structure thins and is replaced by the inward growing radial channels. For UV irradiation at 30 cm fiber travelling distance, the radial channels extend 25 µm into the membrane and a coarse structure with pore sizes >10 µm is found between the surface layers (Fig. 2a). For UV irradiation at fiber travelling distances >30 cm, the same membrane architecture is found.

These findings provide direct observation of the dynamics of STRIPS membrane formation. Recall that phase separation is initiated by ethanol flux to both the external and bore phases. This process results in the formation of a bicontinuous porous structure on the surface of the membrane stabilized via interfacial jamming of nanoparticles. Radial channels, similar to those found in NIPs membranes, also form, stabilized by UV irradiation. The formation of a macroporous structure in the middle of the cross-section (shown in blue in Fig. 2a) can be attributed to the precipitation of poly(HDA) upon instantaneous polymerization. This annular band experiences a delayed ethanol flux and is comprised of a homogeneous liquid mixture prior to polymerization, as demonstrated by comparing the middle layer morphology to the structure resulting from direct UV irradiation of a homogeneous ternary liquid mixture (Supplementary Note 4). A qualitatively different morphology observed in the annular band formed via UV irradiation at >30 cm (pink) likely suggests some type of phase separation took place in this region. The finding that for polymerization distances larger than 30 cm no further changes in morphology can be observed (Supplementary Note 2) indicates that here all water/HDA structures within the fiber have been arrested by interfacially jammed nanoparticles.

The rapid polymerization also reveals the details of the fiber surface structure evolution. Figure 2b shows that the outer surface pores decrease in size as the bijel is polymerized further downstream. UV irradiation at 5 cm fiber travelling distance results in the formation of a nodular scaffold. The magnified inset of Fig. 2b shows clearly that the scaffold is covered with densely-packed silica nanoparticles. Interestingly, at a fiber travelling distance of 15 cm, the nodular structures start to disappear until the micron-sized surface pores completely vanish for UV irradiation at fiber travelling distances larger than 30 cm. The magnified inset shows that here the entire surface is covered with silica nanoparticles. In contrast to the SEM micrographs, confocal microscopy shows that the size and organization of the nodular poly(HDA) structures do not change for different UV-irradiation locations along the fiber trajectory (Supplementary Fig. 2). This finding indicates that the space in the surrounding water channels has been filled with excess nanoparticles. Moreover, it also indicates that phase separation is arrested on the fiber surface by jamming of nanoparticles on the interface before the fiber has travelled 5 cm from the nozzle. We also find that the initial surface pore size (at 5 cm fiber travelling distance) can be tuned by increasing the concentration of the nanoparticles in the membrane (Fig. 2c). For 0.26 g_silica_/g_dry fiber_ average pore size is around 3 µm, whereas for 0.38 g_silica_/g_dry fiber_ the average pore size becomes about 500 nm. This finding indicates that higher silica concentrations lead to earlier arrest of the phase separation on the membrane surface. Analogous to bijels formed by binary phase separation, plotting surface pore size against the inverse silica particle volume fraction yields a linear relationship for sufficiently high particle concentrations (Supplementary Note 3). Moreover, this trend can be altered by increasing CTAB concentration, reducing the surface pore size at a given nanoparticle concentration^34^.

These observations suggest that the membrane surface structures evolve via a two-step process. First, surface pores are formed by arresting phase separation via interfacial nanoparticle jamming. Second, excess nanoparticles accumulate within the surface pores where they also jam and aggregate; we hypothesize that these excess nanoparticles are swept into these pores by the radial mass transfer of ethanol. The final surface pores resulting from this effect determine the flux through the membrane. They are given by the interstices between the nanoparticles, which are small enough (likely <5 nm) to introduce ultrafiltration characteristics to the membranes as will be discussed below.

The final surface pore size and membrane structure can be further tuned by varying the amount of ethanol in the continuous water phase, which we demonstrate with fibers that are polymerized after complete phase separation (~10 min after their extrusion). Figure 2d shows the fiber surface morphologies for different ethanol concentrations in the continuous water phase. The surface pore size increases significantly when the ethanol concentration is raised to 20 vol%. We believe this trend is affected by the aggregation behavior of the silica nanoparticles. In aqueous dispersions, silica nanoparticles aggregate above CTAB concentrations of 0.7–1.0 mM. However, the addition of ethanol shifts the CTAB concentration that induces SiO_2_ nanoparticle aggregation to higher values. This effect results in reduced deposition of nanoparticles into the pores and more porous fiber surfaces.

### Separation properties of STRIPS bijel-derived hollow fiber membranes

**Fig. 3:**
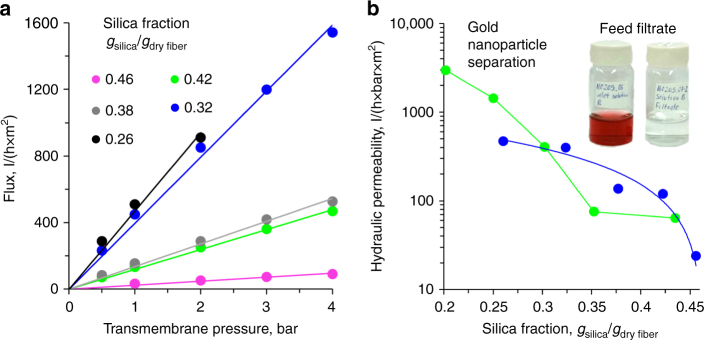
Separation properties of STRIPS bijel-derived membranes. **a** Flux of pure water as a function of transmembrane pressure for membranes polymerized 5 cm from the nozzle with different silica nanoparticle fractions (40 mM CTAB, 0% EtOH in continuous phase). **b** Hydraulic permeability (i.e., slope of lines in **a**) as a function of silica nanoparticle concentration for membranes polymerized at distances of 5 cm (blue points) and 30 cm (green points) from the extrusion nozzle (both 40 mM CTAB, 0% EtOH in continuous phase, curves are drawn to guide the eye). The inset shows photographs of a suspension of citrate stabilized gold nanoparticles before (feed, 0.01 wt% 20 nm Ag particles at pH 8) and after (filtrate) flowing through the membrane fabricated with 0.35 g_silica_/g_dry fiber_, 40 mM CTAB, polymerized at 30 cm distance from the nozzle. UV Vis spectra of the feed and permeate solutions are provided in Supplementary Fig. 1

Using a small-scale hollow fiber membrane module, we characterize the separation properties of STRIPS bijel-derived membranes (Supplementary Methods). The flux through the membranes depends linearly on the transmembrane pressure (Fig. 3a). Furthermore, the flux depends strongly on the silica nanoparticle concentration in the original ternary mixture. In general, increasing the nanoparticle concentration reduces the water flux at a given transmembrane pressure for membranes prepared with UV irradiation for fiber travelling distances of 5 and 30 cm (Fig. 3b). Membranes polymerized at 30 cm fiber travelling distance show significantly larger fluxes for silica fractions <0.3 g_silica_/g_dry fiber_ than do membranes polymerized at 5 cm. On the other hand, above 0.3 g_silica_/g_dry fiber_, both types of membranes show similar fluxes. The fluxes through the membranes may be related to their surface and internal structures (Fig. 2a, b). The hydraulic resistance of membranes UV irradiated at 5 cm fiber travelling distance may arise from the macroporous interior structure. The slight decrease of the flux with increasing silica concentration through membranes polymerized at 5 cm between 0.25–0.42 g_silica_/g_dry fiber_ might be related to the decrease of the pore size of the macroporous middle layer (Supplementary Note 4). In contrast, for membranes UV irradiated at 30 cm fiber travelling distance, the major resistance is attributed to the nanoparticle-filled surface pores. The linearity of the flux as a function of pressure indicate that the silica nanoparticle packings filling the surface pores withstands pressures of up to 4 bar. The determination of the precise roles of different regions in determining the transport properties of the bijel-derived membranes is the focus of ongoing research.

STRIPS bijel-derived membranes formed by UV irradiation >30 cm fiber travelling distance can filter 20 nm gold nanoparticles when they have silica fractions larger than 0.35 g_silica_/g_dry fiber_ (dead-end filtration test, see inset Fig. 3b, and Supplementary Methods and Supplementary Note 1). The corresponding membrane has a hydraulic permeability of 70 l h^−1^ m^−2^ bar^−1^, comparable to the values reported for typical ultrafiltration membranes made via NIPS^37^. Based on the surface morphology of this membrane (Figs. 2c and 3), we believe the dense packing of nanoparticles on the surface functions as the selective layer that enables the separation of gold nanoparticles. This reasoning is supported by the finding that membranes polymerized at 5 cm fiber traveling distance, which lack such a densely packed layer, could not filter the gold nanoparticles at any silica fraction. From this finding, we also conclude that the pore size of the macroporous structure in the middle of these fibers is too large for gold nanoparticle filtration.

### Functional hollow fiber membranes

**Fig. 4:**
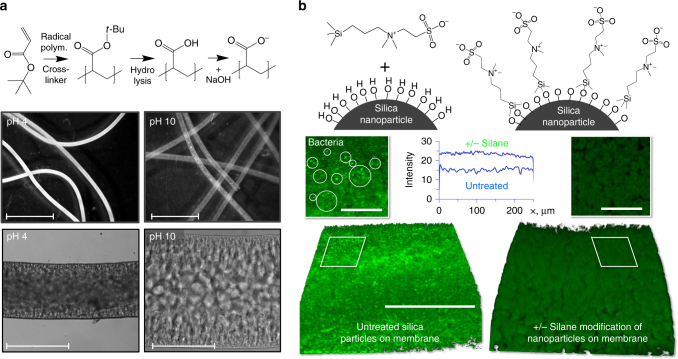
Additional features of STRIPS-bijel derived membranes. **a** Formation of pH-responsive poly(acrylic acid) hydrogel fibers by hydrolysis of fibers composed of HDA cross-linked poly(*tert*-butylacrylate). The photo- and micro- graphs show the fibers unswollen at pH 4 and swollen at pH 10. Scale bars upper micrographs 2 mm, scale bar lower micrographs 200 µm. **b** Zwitterionic silane functionalization of the densely-packed silica nanoparticle surface of the membranes. Three dimensional confocal micrographs show that the unmodified membranes (having a faint green fluorescence themselves) are partially coated by bright speckles of green-fluorescent *Streptococcus* mutants (see inset), while the zwitterionic silane-functionalized membrane does not show significant bacteria adhesion. The inset diagram shows the comparison of the average fluorescence intensity along the length of both membranes. Both membranes have been imaged at the same settings for confocal micrograph acquisition. Scale bar isometric membrane perspective 50 µm, scale bar magnified insets 20 µm

The fabrication of hollow fiber membranes using STRIPS bijels presents a unique opportunity to create functional membranes. For example, pH-responsive nanocomposite/hydrogel membranes can be fabricated by employing a hydrophobic monomer that can be subsequently converted to a hydrophilic and pH-responsive repeat unit. We demonstrate this capability by using a mixture of 10 wt% hexanedioldiacrylate (HDA) in *tert*-butylacrylate. After UV-induced polymerization, we hydrolyze a HDA cross-linked poly(*tert*-butylacrylate) fiber in a mixture of trifluoroacetic acid and formic acid^38^ to create the HDA cross-linked poly(acrylic acid) hydrogel fiber shown in Fig. 4a. These bijel-derived hollow fiber membranes undergo dramatic swelling in a basic solution (Fig. 4a). The water swollen fiber also has enhanced flexibility. These stimuli-responsive fibers could potentially be used as a pH-responsive smart membranes that open/close their surface pores in response to changes in the solution pH^39^.

An important feature unique to STRIPS bijel-derived membranes is the dense silica nanoparticle layer present on the surface, which provides a simple and versatile route for membrane surface functionalization by silanization. This may provide a route to circumvent the major challenge presented by biofouling^40^ in membrane-based water treatment. Bacteria colonize membrane surfaces to form biofilms, with negative implications for membrane performance and water safety. Inspired by recent reports that show surfaces densely covered with zwitterionic functional groups can prevent adhesion of bacteria^41, 42^, we functionalize silica nanoparticles on the membrane with a zwitterionic silane (sulfobetaine silane, SBSi), and test bacteria adhesion on such membranes. Figure 4b shows that the unmodified membrane surface is covered with fluorescent bacteria after incubation in bacteria suspension for 16 h, whereas few bacteria can be observed on the SBSi-modified membrane, indicating that the surface modification has indeed imparted anti-biofouling properties. This is but one example of the broad potential of membrane surface functionalization for STRIPS-bijel membranes.

In conclusion, we have introduced an approach for the fabrication of functional separation membranes with dense nanoparticle surface layers based on STRIPS. Nanoparticle-decorated membranes with a macroporous surface pore structure, or with a nanoparticle-filled surface pores can be fabricated. STRIPS allows for the use of highly cross-linkable monomers to manufacture hollow fiber membranes and also allows for tunable structures. By changing the monomer, we also demonstrate that pH-responsive hydrogel membranes can be prepared. Furthermore, our method facilitates the membrane functionalization via silanization of the dense layer of silica nanoparticles on the membrane surface. While this study used silica nanoparticles and simple monomers for structure formation, introduction of catalytic or mesoporous nanoparticles as well as functional monomers will lead to generation of multifunctional nanocomposite membranes with functionality designed for advanced separations applications.

## Methods

### Preparation of liquid mixtures

A concentrated silica nanoparticle dispersion (Ludox TMA) in ethanol is prepared: 1 M HCl is added to reduce the pH value to pH 3. Dispersion of 100 ml is transferred into a dialysis bag (MW cutoff = 4000). The dialysis bag is immersed in a beaker containing 500 ml ethanol (190 proof). After 12 and 24 h, respectively, the ethanol is replaced by 500 ml ethanol (200 proof). Dialysis concentrates the silica nanoparticles to 57.8 wt% in ethanol (density 1.206 g ml^−1^) (solution A). The following stock solutions are employed: (solution B) 200 mM CTAB in ethanol (200 proof), (solution C) 48.6 wt% silica nanoparticles in water (density 1.32 g ml^−1^) at pH 3 (obtained by evaporating parts of the water in the original Ludox TMA dispersion), (solution D) HDA (99% reactive esters, Alfa Aesar), (solution E) 2-hydroxy-2-methylpropiophenone (97%, Sigma-Aldrich). To prepare 10 ml of a ternary fluid producing a bijel membrane with 0.477 g_silica_/g_dry fiber_, we mix 3.5 g solution A, 1.6 g solution B, 2.7 g solution C, 3.64 g solution D, and 0.2 g solution E and mix vigorously. For this mixture, we calculate 0.477 g_silica_/g_dry fiber_ based on the assumption that all silica particles remain in the fiber and that the dry fiber is composed of the mass of poly(HDA) and the silica in solution A and solution C. Here: (1.77 g silica + 1.56 g silica) / (1.77 g silica + 1.56 g silica + 3.64 g poly(HDA)).

If fibers with lower silica concentrations are desired, smaller volumes of solution A and/or C are added, with the volume reduction being replaced by a corresponding volume of pure ethanol or water. Bore fluid: we dissolve 1 wt% of polyethyleneglycol (600 kDa) and 0.1 wt% methylene blue in water (methylene blue facilitates the detection of defects on the membrane during formation). Continuous outer phase (nonsolvent bath): we use municipal tap water (Philadelphia, PA, USA) and adjust the pH to 3 by adding 1.0 M HCl.

### Fiber extrusion

Before extrusion, a solution composed of 0.5 wt% poly(diallyldimethylammonium chloride) (polyDADMAC) with a molecular weight of 200–300 kDa and 0.5 mol l^−1^ NaCl is allowed to flow through the channels in order to coat the glass capillaries with polyDADMAC to prevent fiber adhesion. Three syringe pumps (Harvard PHD Ultra, New Era NE-300) are loaded with plastic syringes containing the ternary fluid, bore fluid, and outer water phase, and connected with PTFE tubing to the fiber extrusion device. The extrusion device is held vertically by finger clamps attached to a lab stand (Supplementary Fig. 14a). The fiber is extruded at the flow rates for the outer water *Q*_W_ of 0.5–2 ml min^−1^, ternary fluid *Q*_T_ ranging from 10–20 ml h^−1^, bore fluid *Q*_B_ ranging from 1 to 10 ml h^−1^.

### Hydrogel formation

The same solutions as in section “Preparation of liquid mixtures” are employed, except solution D is replaced by a mixture of tertiary-butylacrylate with 10 wt% HDA. Then the following ternary mixture with a volume of 5 ml is prepared: 2.16 g of solution A, 0.96 g of solution B, 1.525 g of solution C, 1.15 g of solution D, and 0.07 g of solution E. For this work, we have extruded the fiber without a bore fluid. After UV polymerization, the fiber is immersed in a mixture of 20 wt% formic acid in trifluoroacetic acid for 12 h. The acid mixture is replaced three times with ethanol and then with water. After the addition of sodium hydroxide, the swelling of the fiber can be observed.

### Bacteria adhesion test

We synthesize the SBSi as described in the literature^2^. The fibers are washed as described under fiber post treatment and thereafter are immersed in a solution composed of 20 mmol l^−1^ of SBSi, 0.2 vol% of water, 0.3 vol% acetic acid, and the remainder ethanol, and placed in a heating bath at 70°C for 12 h. The fibers are then washed with ethanol and subsequently in phosphate buffer at pH 7.4. The fibers are then immersed in ultrafiltered tryptone-yeast extract broth with 1% glucose are with Gfp-labeled *Streptococcus mutans* bacteria for 16 h under gentle shaking at 37 °C. After rinsing the fibers with the phosphate buffer, they are directly transferred for visualization in a confocal microscope.

### Data availability

Data available from authors on request.

## Electronic supplementary material

Supplementary Information

## References

[1] Sholl DS, Lively RP (2016). Seven chemical separations to change the world. Nature.

[2] Shannon MA (2008). Science and technology for water purification in the coming decades. Nature.

[3] Lively RP, Sholl DS (2017). From water to organics in membrane separations. Nat. Materials.

[4] Tiraferri A, Kang Y, Giannelis EP, Elimelech M (2012). Superhydrophilic thin-film composite forward osmosis membranes for organic fouling control: fouling behavior and antifouling mechanisms. Environ. Sci. Technol..

[5] Ma Y (2012). Preparation and characterization of PSf/clay nanocomposite membranes with LiCl as a pore forming additive. Desalination.

[6] Wu H, Tang B, Wu P (2014). Development of novel SiO 2–GO nanohybrid/polysulfone membrane with enhanced performance. J. Memb. Sci..

[7] Worthley CH, Constantopoulos KT, Ginic-Markovic M, Markovic E, Clarke S (2013). A study into the effect of POSS nanoparticles on cellulose acetate membranes. J. Memb. Sci..

[8] Anadão P, Sato LF, Wiebeck H, Valenzuela-Díaz FR (2010). Montmorillonite as a component of polysulfone nanocomposite membranes. Appl. Clay Sci..

[9] Mauter MS (2011). Antifouling ultrafiltration membranes via post-fabrication grafting of biocidal nanomaterials. ACS Appl. Mater. Interfaces.

[10] Ngang H, Ooi B, Ahmad A, Lai S (2012). Preparation of PVDF–TiO_2_ mixed-matrix membrane and its evaluation on dye adsorption and UV-cleaning properties. Chem. Eng. J..

[11] Gohari RJ, Lau WJ, Matsuura T, Halakoo E, Ismail AF (2013). Adsorptive removal of Pb (II) from aqueous solution by novel PES/HMO ultrafiltration mixed matrix membrane. Sep. Purif. Technol..

[12] Hoek EM, Ghosh AK, Huang X, Liong M, Zink JI (2011). Physical–chemical properties, separation performance, and fouling resistance of mixed-matrix ultrafiltration membranes. Desalination.

[13] Jeon MY (2017). Ultra-selective high-flux membranes from directly synthesized zeolite nanosheets. Nature.

[14] Zodrow K (2009). Polysulfone ultrafiltration membranes impregnated with silver nanoparticles show improved biofouling resistance and virus removal. Water Res..

[15] Ben-Sasson M (2013). Surface functionalization of thin-film composite membranes with copper nanoparticles for antimicrobial surface properties. Environ. Sci. Technol..

[16] Gao P, Liu Z, Tai M, Sun DD, Ng W (2013). Multifunctional graphene oxide–TiO_2_ microsphere hierarchical membrane for clean water production. Appl. Catal. B Environ..

[17] Yin J, Deng B (2015). Polymer-matrix nanocomposite membranes for water treatment. J. Memb. Sci..

[18] Alhoshan M, Alam J, Dass LA, Al‐Homaidi N (2013). Fabrication of polysulfone/ZnO membrane: influence of ZnO nanoparticles on membrane characteristics. Adv. Polym. Technol..

[19] Yan L, Hong S, Li ML, Li YS (2009). Application of the Al 2 O 3–PVDF nanocomposite tubular ultrafiltration (UF) membrane for oily wastewater treatment and its antifouling research. Sep. Purif. Technol..

[20] Soroko I, Livingston A (2009). Impact of TiO_2_ nanoparticles on morphology and performance of crosslinked polyimide organic solvent nanofiltration (OSN) membranes. J. Memb. Sci..

[21] Sun M, Su Y, Mu C, Jiang Z (2009). Improved antifouling property of PES ultrafiltration membranes using additive of silica−PVP nanocomposite. Ind. Eng. Chem. Res..

[22] Escobar-Ferrand L, Li D, Lee D, Durning CJ (2014). All-nanoparticle layer-by-layer surface modification of micro and ultrafiltration membranes. Langmuir.

[23] Chan EP (2014). Tailoring the permselectivity of water desalination membranes via nanoparticle assembly. Langmuir.

[24] Jadav GL, Aswal VK, Singh PS (2010). SANS study to probe nanoparticle dispersion in nanocomposite membranes of aromatic polyamide and functionalized silica nanoparticles. J. Colloid Interface Sci..

[25] Teow Y, Ahmad A, Lim J, Ooi B (2012). Preparation and characterization of PVDF/TiO_2_ mixed matrix membrane via in situ colloidal precipitation method. Desalination.

[26] Vanherck K, Vandezande P, Aldea SO, Vankelecom IF (2008). Cross-linked polyimide membranes for solvent resistant nanofiltration in aprotic solvents. J. Memb. Sci..

[27] Herzig E, White K, Schofield A, Poon W, Clegg P (2007). Bicontinuous emulsions stabilized solely by colloidal particles. Nat. Mater..

[28] Tavacoli, J. W., Thijssen, J. H. & Clegg, P. S. in *Particle-Stabilized Emulsions and Colloids* (eds Ngai, T. & Bon, S.) Ch. 6, 129–168 (RSC Publishing, 2014).

[29] Lee MN, Mohraz A (2010). Bicontinuous macroporous materials from bijel templates. Adv. Mater..

[30] Chung H, Ohno K, Fukuda T, Composto RJ (2005). Self-Regulated Structures in Nanocomposites by Directed Nanoparticle Assembly. Nano Lett...

[31] Guillen GR, Pan Y, Li M, Hoek EM (2011). Preparation and characterization of membranes formed by nonsolvent induced phase separation: a review. Ind. Eng. Chem. Res..

[32] Smolders C, Reuvers A, Boom R, Wienk I (1992). Microstructures in phase-inversion membranes. Part 1. Formation of macrovoids. J. Memb. Sci..

[33] Loeb S, Sourirajan S (1962). Sea water demineralization by means of an osmotic membrane. Adv. Chem..

[34] Haase MF, Stebe KJ, Lee D (2015). Continuous fabrication of hierarchical and asymmetric bijel microparticles, fibers, and membranes by solvent transfer‐induced phase separation (STRIPS). Adv. Mater..

[35] Haase MF, Sharifi-Mood N, Lee D, Stebe KJ (2016). In situ mechanical testing of nanostructured bijel fibers. ACS Nano.

[36] Yao J, Wang K, Ren M, Liu JZ, Wang H (2012). Phase inversion spinning of ultrafine hollow fiber membranes through a single orifice spinneret. J. Memb. Sci..

[37] Mehta A, Zydney AL (2005). Permeability and selectivity analysis for ultrafiltration membranes. J. Memb. Sci..

[38] Tu F, Lee D (2014). Shape-changing and amphiphilicity-reversing Janus particles with pH-responsive surfactant properties. J. Am. Chem. Soc..

[39] Lee D, Nolte AJ, Kunz AL, Rubner MF, Cohen RE (2006). pH-induced hysteretic gating of track-etched polycarbonate membranes: swelling/deswelling behavior of polyelectrolyte multilayers in confined geometry. J. Am. Chem. Soc..

[40] Nguyen T, Roddick FA, Fan L (2012). Biofouling of water treatment membranes: a review of the underlying causes, monitoring techniques and control measures. Membranes.

[41] Yeh S-B, Chen C-S, Chen W-Y, Huang C-J (2014). Modification of silicone elastomer with zwitterionic silane for durable antifouling properties. Langmuir.

[42] Knowles BR, Wagner P, Maclaughlin S, Higgins MJ (2017). Silica nanoparticles functionalized with zwitterionic sulfobetaine siloxane for application as a versatile antifouling coating system. ACS Appl. Mater. Interfaces.

