# Supplementary material for: Multifunctional nanocomposite hollow fiber membranes by solvent transfer induced phase separation
Source: Nat Commun. 2017 Nov 1;8:1234. doi: 10.1038/s41467-017-01409-3 (PMC5663856; doi:10.1038/s41467-017-01409-3)
Supplement: Supplementary file 1 — Supplementary Information [file 41467_2017_1409_MOESM1_ESM.pdf]

Supplementary Note 1: Measurement of gold nanoparticle separation

We synthesize two different batches of gold nanoparticles by reducing gold chloride (HAgCl) with sodium citrate.[3] 50 ml of an aqueous solution containing 0.01 wt-% of HAgCl is heated to boiling temperature. 1 ml of a 1 wt-% solution of sodium citrate is added to the boiling liquid to synthesize gold nanoparticles with approximate diameters of 20 nm.

We employ the flux testing device shown in Supplementary Figure 5 to test the separation of gold nanoparticles. The gold nanoparticle suspension is introduced through the feed channel into the center chamber. At a feed pressure of 2 bars we collect the filtrate and measure the UV-vis spectrum of the filtrate. Supplementary Figure 1 shows the UV-vis spectra of a 20 nm gold nanoparticle suspension before and after filtration through a membrane with 0.35 $g_{silica}/g_{dry\text{-}fiber}$ (polymerization distance 30 cm).

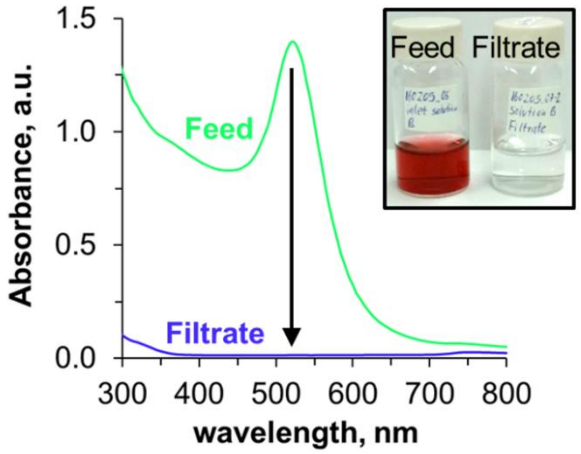

Supplementary Figure 1: *Separation of gold nanoparticles.* UV/vis spectra of feed and filtrate for gold nanoparticles of 20 nm size.

The UV/vis spectra of the feed suspension shows a peak at 510 nm, while this peak completely disappears for the filtrate, indicating removal of the gold nanoparticles by the membrane.

Supplementary Note 2: Confocal microscopy of STRIPS-bijel fibers

Figure 2 shows that the surface of fibers UV-irradiated at distances > 30 cm are covered by a densely-packed layer of silica nanoparticles. We employ confocal microscopy to visualize the poly(HDA) structures corresponding to this state. To this end, we add the water insoluble dye Nile Red to the ternary mixture used for fiber fabrication. The fibers are collected on the bottom of the rotating water cylinder. Next, we transfer the fibers (immersed in water) to the stage of a confocal microscope (Thorlabs confocal laser

scanner paired with Olympus IX73 microscope). Supplementary Figure 2a shows a 3-dimensional reconstruction of the fiber surface obtained by image processing (ImageJ) of a confocal z-stack.

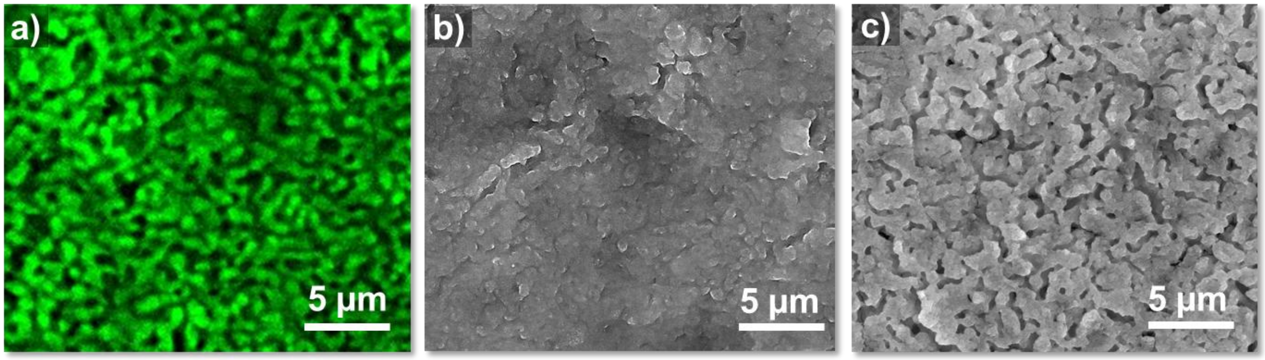

Supplementary Figure 2: a) Confocal laser scanning micrograph of the surface of a fiber formed with the same composition as the fiber in Supplementary Figure 2a,b that has not been polymerized. Green regions correspond to liquid HDA dyed with Nile Red, black regions to water pores. **b)** Scanning electron micrograph (SEM) of a STRIPS-bijel fiber surface, polymerized at 30 cm fiber travelling distance, **c)** SEM of a STRIPS bijel fiber surface, polymerized at 5 cm fiber travelling distance.

The confocal micrograph shows that, despite the layer of densely packed silica nanoparticles (shown in Supplementary Figure 2b), a nodular poly(HDA) scaffolds can be found. This finding shows that the water pores on the surface of the bijel derived hollow fiber membrane are filled with excess silica nanoparticles.

Moreover, this finding also demonstrates that the surface structures do not coarsen after 5 cm fiber travelling distance. Supplementary Figure 2c shows the polymerized nodular surface scaffold of a fiber polymerized at this distance. A comparison of the confocal microscopy of the unpolymerized STRIPS bijel fiber in Supplementary Figure 2a and the electron microscopy in Supplementary Figure 2c shows the same organization and dimensions of the nodular surface scaffold. This indicates the structural arrest of the liquid HDA/water scaffold by nanoparticle jamming.

We also find that the internal structures (radial channels) of the STRIPS-bijel are arrested by interfacial nanoparticle jamming before polymerization. Supplementary Figure 3 shows a confocal microscopy z-stack of the internal structures of an unpolymerized STRIPS-bijel fiber (formed without bore fluid).

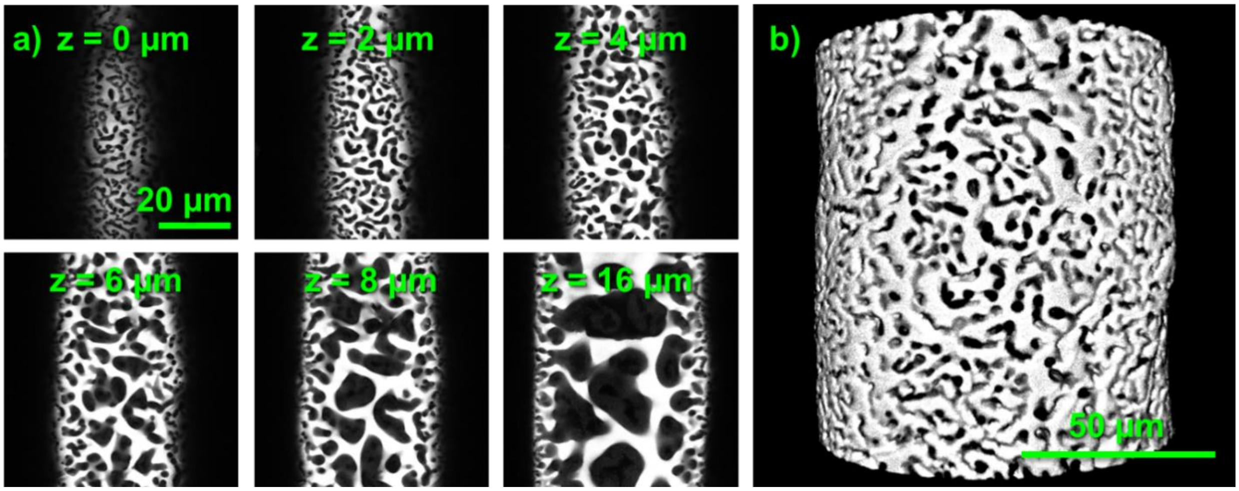

Supplementary Figure 3: Confocal microscopy of an unpolymerized STRIPS bijel fiber. White areas correspond to liquid HDA, black areas to liquid water. **a)** "Tomography" or z-stack of a section of the fiber, slices of fluorescence light information for different distances from the fiber surface (z in µm). **b)** 3D reconstruction of the z-stack.

Supplementary Note 3: Surface pore size dependence on nanoparticle volume fraction and CTAB concentration

The first experimental work on bijels[5] showed that for high and intermediate concentrations of surface active colloids (interfacial stabilizers) a linear relationship between the pore size (typical separation distance of bicontinuous domains) and the inverse volume fraction of the colloids $1/\Phi$ exists. For low concentrations of colloids, or high $1/\Phi$ the linear relationship breaks down due to the formation of nonuniform structures.

A slope of 0.72 µm for 290 nm silica particles was reported; this slope was compared to that expected for spherical droplets covered by a close packed monolayer of particles ($\xi = \pi / \sqrt{3})(d / \Phi)$, for which the expected value is 1.05 µm. Lower values for the slope indicate non-close packing, suggesting that these bijels were stabilized by a non-close packed monolayer of particles. This was indeed confirmed experimentally by high magnification microscopy.

We also find a linear relationship of the surface pore size of STRIPS-bijels on the inverse volume fraction $1/\Phi$ of the silica nanoparticles (20 nm, Supplementary Figure 4). For 80 mM CTAB in the ternary mixture, the prefactor 0.146 is larger than what would be expected for close packing of particles at the interface (0.036), potentially suggesting that there are multiple layers of particles at the oil-water interface.

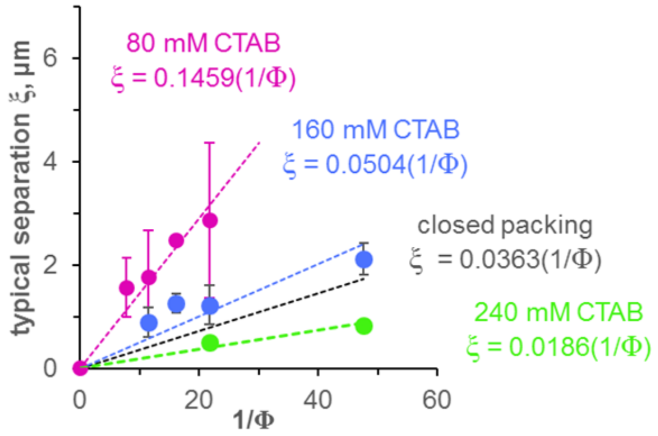

Supplementary Figure 4: Relationship between STRIPS-bijel surface pore size (ξ, typical separation) and inverse volume fraction $1/\Phi$ of 20 nm silica nanoparticles for different CTAB concentrations. Also shown is the hypothetical relationship for densely packed 20 nm particles on spherical droplets of size ξ.

For 160 mM and 240 mM CTAB we find that the linear slope decreases, indicating more efficient particle jamming on the oil/water interface. These findings were also described in our previous publication.[4]

Supplementary Note 4: Characterization of macroporous middle layer

To clarify the origin of the middle layer formed at early polymerization distances (5 cm, 15 cm) we perform an additional experiment. A ternary mixture with silica nanoparticles, CTAB and HMPP is spread on a microscope slide and immediately polymerized by UV-light irradiation. The resulting polymer material is washed, dried and transferred to the stage of a scanning electron microscope. Supplementary Figure 10a shows the resulting structure. A macroporous structure with pore sizes ranging from 100 – 650 nm (see pore size distribution in Supplementary Figure 5). The structure is remarkably similar to the structure found in the middle layer of the hollow fiber membrane polymerized at 5 cm distance (Supplementary Figure 10b).

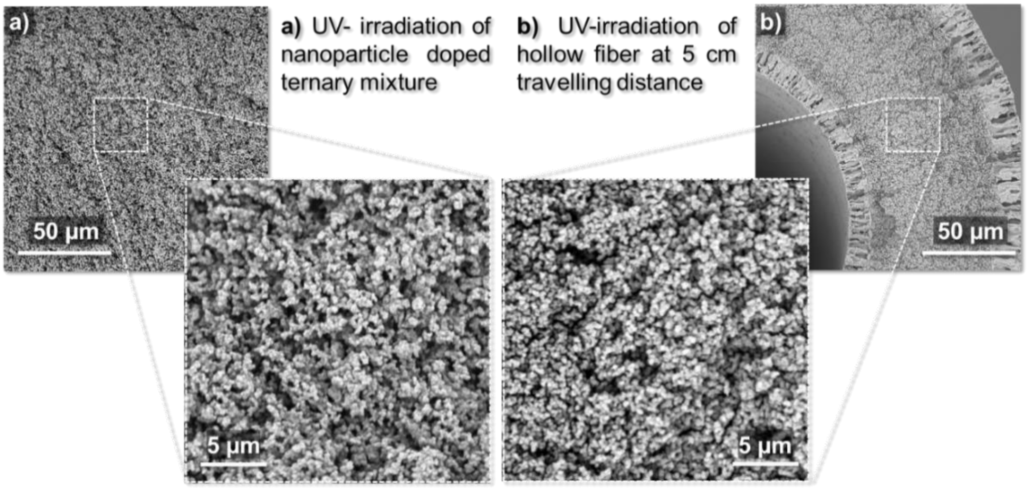

Supplementary Figure 5: Characterization of macroporous middle layer. **a)** Scanning electron micrographs of the macroporous structure formed by rapid UV polymerization of a ternary liquid mixture spread on a microscope slide. **b)** Cross section of fiber UV-irradiated at 5 cm distance.

We conclude that before polymerization at 5 cm polymerization distance the middle layer was still a homogeneous ternary liquid mixture. UV-polymerization results in the formation of a macroporous structure. We measure the pore size distribution of the macroporous middle layer by image analysis of the scanning electron micrograph (Supplementary Figure 6). The pore size weakly dependent on the silica concentration: Increasing silica concentrations reduce the pore size.

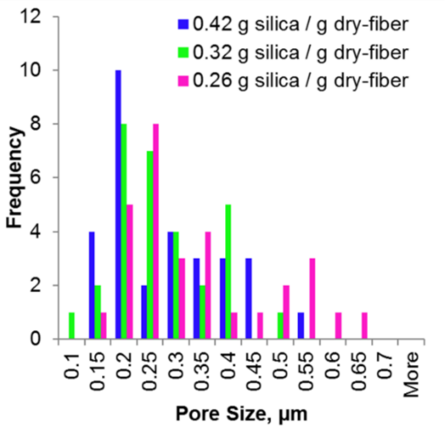

Supplementary Figure 6: Histograms of pore size distribution of the middle layer. Pore size distribution was measured by analysis of scanning electron micrographs. The average pore size is 200 nm with a maximum of 650 nm and a minimum of 100 nm.

Supplementary Note 5: Confocal microscopy of fiber extruded in continuous water phase with 20 vol-% ethanol

We perform confocal microscopy of the STRIPS bijel structure formed at 20% ethanol prior to polymerization (Supplementary Figure 7) and find the same morphology as the one in the scanning electron micrograph of Figure 2d, indicating that phase separation took place and led to the observed bicontinuous surface structure.

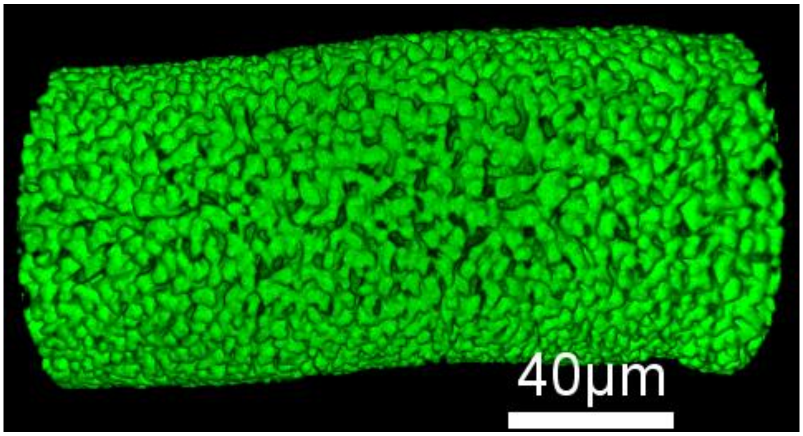

Supplementary Figure 7: Confocal microscopy of an unpolymerized STRIPS bijel fiber formed in a continuous water phase containing 20% ethanol. Green regions correspond to liquid HDA, black regions to liquid water. Nanoparticles are not visible in the confocal micrograph.

Supplementary Note 6: Bursting/Bulging of Fibers and Heterogeneous Surface Structures

During the formation of fibers with thin walls (e.g. for flow rates Q_W 1 ml/min, Q_T 10 ml/hr, Q_B 15 ml/hr) and high nanoparticle concentrations (silica fraction > 0.32 $g_{silica}/g_{dry\text{-}fiber}$) periodic bursting (or rupture) can be observed (Supplementary Figure 8). We add the dye methylene blue to the bore fluid to render this effect visible. After a typical travelling distance of the fiber of 15 cm blue colored fluid emerges locally on the surface of the fiber. Two rupture sites have typically periodic spacing, but this spacing can range in separate experiments from 2 cm up to 20 cm.

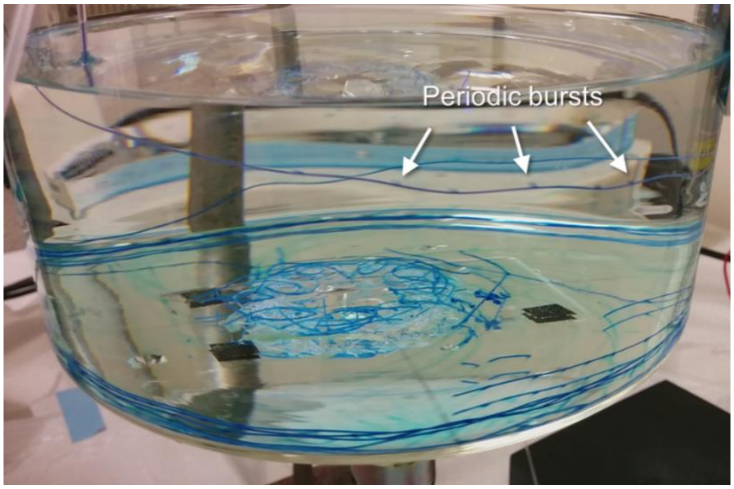

Supplementary Figure 8: *Fiber bursting made visible by dying bore fluid*. **a)** After exiting the extrusion nozzle, the fiber travels for 10 – 15 cm without signs of bursting. However, at 10 – 15 cm the leakage of blue dye from the ruptured fiber can be observed (in this example with a periodic spacing of approx. 2 cm.).

Bursting occurs more frequently for high ratios of Q_B–to-Q_T. However, it is not exclusively determined by the flow-rates, since we observe that for the same set of flow rates periodic bursting can take place in one experiment, but not during another. An effective method to prevent bursting is to carry out the UV-radiation of the fiber at 5 cm. This is most likely a result of the enhancement of the mechanical strength of the fiber wall after polymerization, suppressing bursting.

Scanning electron micrographs in Supplementary Figure 9 reveal that the bursting sites are longitudinal cracks in the fiber wall.

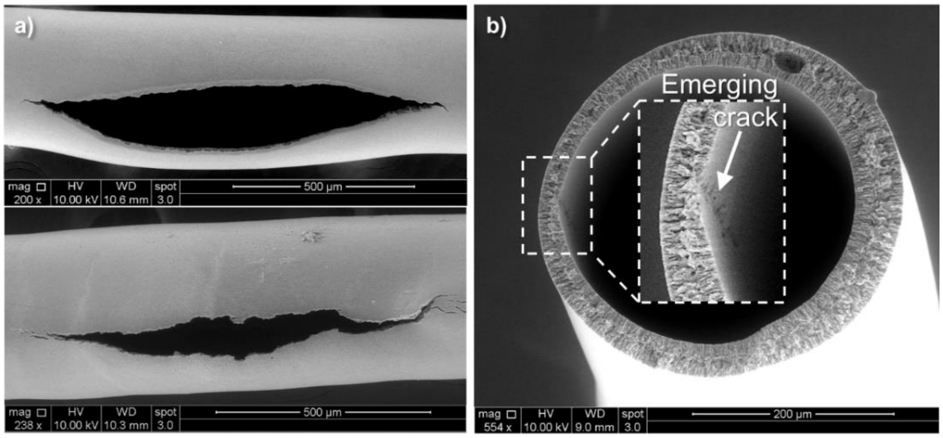

Supplementary Figure 9: Scanning *electron micrographs of fiber defects*. **a)** Examples of longitudinal cracks formed during fiber extrusion. **b)** Cross-section of a hollow fiber with off centered bore channel. Magnified area shows the narrowest section of the fiber wall with onset of a bursting site.

We find cracks resembling those of failed steel vessels under internal pressure where the circumferential stress leads to longitudinal cracks. We hypothesize that the ethanol diffusing into the bore channel is the cause for the internal pressure. The circumferential stress caused by the internal pressure exceeds the mechanical strength of the fiber wall resulting in bursting. For fibers with off-centered bore channel (Supplementary Figure 9b) bursting takes place where the wall is the thinnest. Our hypotheses are supported by the finding that a reduction of the ethanol and water concentrations in the ternary fluid mixture reduces the tendency of fiber bursting. Furthermore, we find that with well aligned coaxial extrusion nozzles bursting is minimized as well.

For fibers with thick walls (e.g. for flow rates Q_W 1 ml/min, Q_T 15 ml/hr, Q_B 2 ml/hr) and high nanoparticle concentrations (silica fraction > 0.32 $g_{silica}/g_{dry\text{-}fiber}$), periodic surface bulging can be observed (Supplementary Figure 10). Like fiber bursting, the bulges occur with periodic spacing of about 2 – 4 cm. SEM micrographs show that these regions have larger surface pores likely due to the expansion of the fiber surface. Heterogeneous surface structures can also be found for thick walled fibers (flow rates Q_W 1 ml/min, Q_T 15 ml/hr, Q_B 2 ml/hr). We find that the bulging and heterogeneous surface structures can be significantly suppressed by the addition of ethanol to the continuous water phase in concentrations of 5 – 15 vol% and the reduction of ethanol and water fraction in the initial ternary mixture, suggesting that these structures likely emerge due to very large ethanol gradient that develops during STRIPS. We exclude the effect of gravity favoring upward ethanol diffusion due to its lower density compared to water, because we also observe heterogeneous surface structures for fibers that have been extruded over a vertical distance of 40 cm.

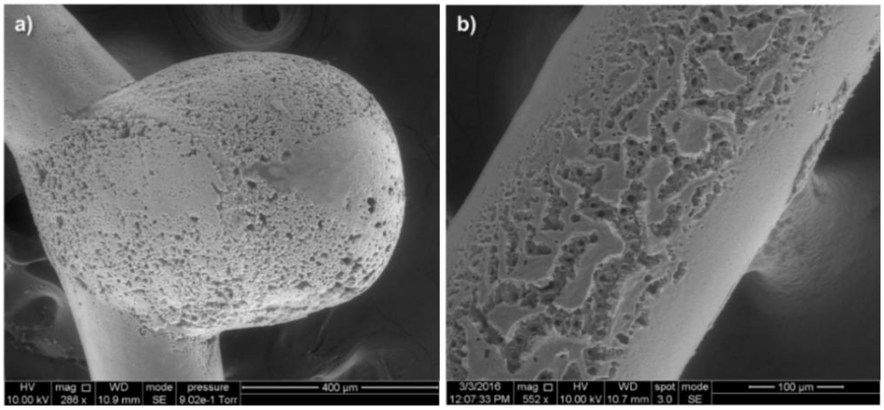

Supplementary Figure 10: *Bulging and surface heterogeneity*. **a)** SEM micrograph of a bulged-out region of a fiber. **b)** SEM micrograph of a fiber with heterogeneous surface structure. The visible side has surface pores with tens of micrometers in size while the opposite side of the fiber is coated with a densely-packed film of silica nanoparticles.

Supplementary Methods

Fiber extrusion device. Glass capillaries are purchased from Vitrotubes®. Four different capillaries are used with the following specifications (round or square refer to the cross-section shape, d_i, d_a and L to inner, outer diameter and total length, respectively): *A* – round, d_i 0.2 mm, d_a 0.29 mm, L 8 cm *B* – square, di 0.3 mm, d_a 0.6 mm, L 3 cm, *C* – round, d_i 0.8 mm, d_a 1 mm, L 4 cm, *D* – square, d_i 1.05 mm, d_a 1.5 mm). See Supplementary Figure 11 for the device assembly process.

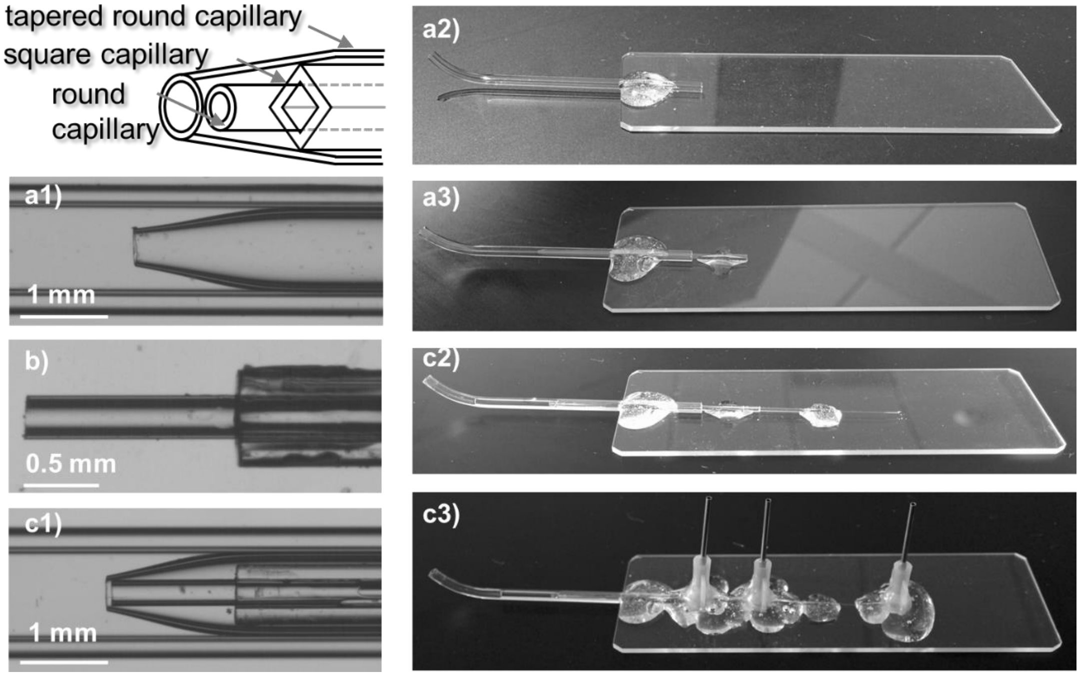

Supplementary Figure 11: Fiber extrusion device assembly. **a1)** micrograph of tapered fiber extrusion nozzle C in D**, a2)** photograph of capillary D glued on microscope slide, **a3)** photograph of capillary C in

D, **b)** micrograph of capillary A in B, **c1)** micrograph of assembled capillaries A in B in C in D, **c2)** photograph of assembled capillaries A in B in C in D, **c3)** photograph of final device with dispensing needle inlets connected to capillaries A, C, D.

C is tapered, cut with a Sutter® Scoring tile and polished with sandpaper to give an orifice of approximately 0.4 mm. *D* is glued on a microscope slide with 5-min epoxy and bent under a flame to give a smooth 45° to 60° bend. *C* is inserted into *D* with the distance between *C* and *D* orifice 1 – 2 cm, and glued with epoxy. The capillary *A* is cut evenly with the tile. *A* is then introduced into *B*. We insert the interdigitated capillaries *A*-*B* into *C* and push *B* all the way inside until it makes contact with the tapered walls of *C*. We then push *A* through *B* and measure with a microscope the required distance for *A* to protrude out of *B* so that the orifices of *A* and *C* are at the same position. *A*-*B* are then taken out of *C* again and *A* is glued inside *B* with Norland Adhesive (NO-81) under UV-light irradiation to provide the measured protruding distance. *A*-*B* is then reinserted into *C* and the orifice of *A* is centered in the orifice of *C* with the help of the microscope (a second microscope is employed to center the capillary in vertical direction via a side-view perspective). *A*-*C* is then mounted to the microscope slide with epoxy. Grooves are cut into the plastic housing of three dispensing needles, which are located on the microscope slide to intersect with the inlets of *A*, *C* and *D*, respectively. The needle plastic housing is then embedded in epoxy glue.

Ternary Mixture preparation

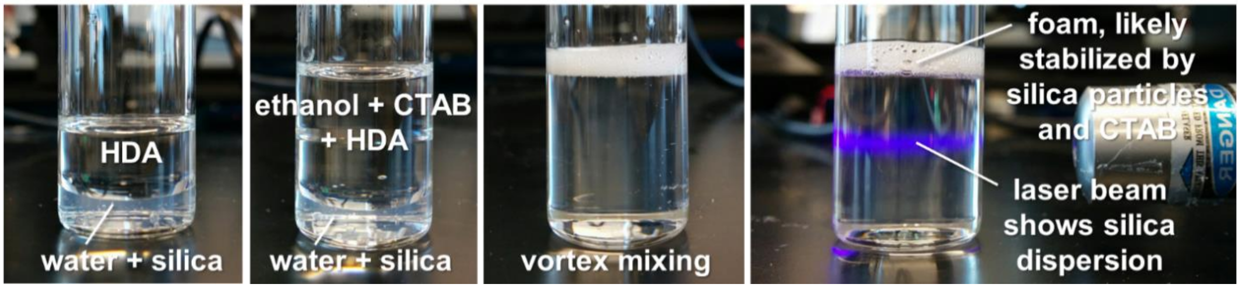

Supplementary Figure 12: Mixing of water, HDA and ethanol (with silica nanoparticles and CTAB as additives).

Fiber extrusion setup and post-treatment. *Turntable setup:* A custom-built turntable is used for rotating the collection bath (Supplementary Figure 13). A thrust needle roller bearing with two washers (MC-Master CARR 5909K22, 5909K94) is placed on two lab-jacks. A laser-cut PMMA ring (d_i 79 mm, d_a 100 mm) holds the bearing and washers in place. From below a DC-gearmotor (McMaster 2709K13) is connected

via a 4 cm D-profile shaft/shaft adapter to a laser cut PMMA plate with a D-profile cutout in the center. The plate has a PMMA ring (d_i 105 mm, d_a 115 mm) attached to the bottom that slips over the ring, surrounding the needle bearing/washer assembly. The motor is connected to a 0 – 18 V AC to DC adjustable transformer.

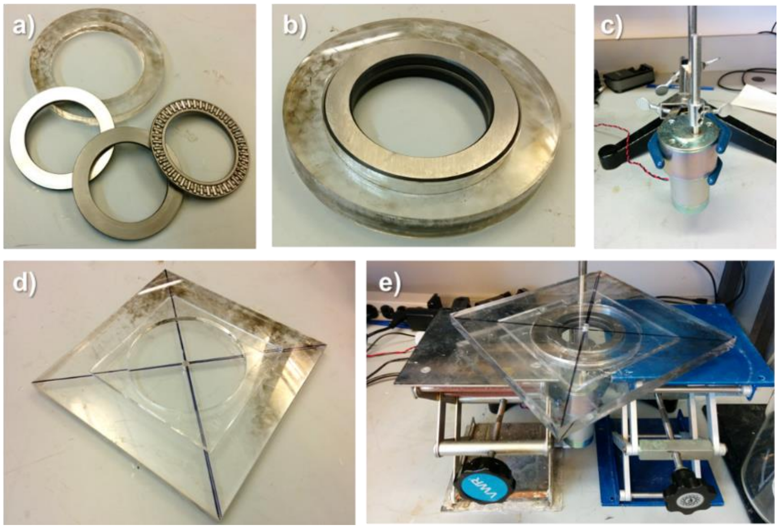

Supplementary Supplementary Figure 13: Turntable setup. **a)** Thrust needle roller bearing with 2 washers and laser-cut PMMA ring **b)** Assembled needle bearing. **c)** DC motor with shaft extension and D-shaft profile. **d)** laser-cut turntable plate with D-shaft profile cutout in the center. **e)** Assembled turntable with lab jacks as supports.

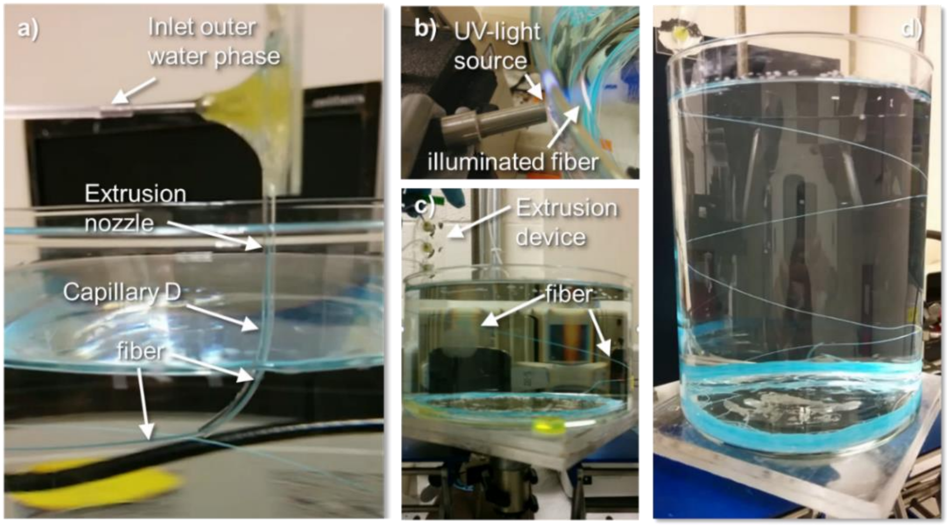

Supplementary Figure 14: *Images of experimental setup for fiber extrusion shown schematically in Figure 1b*. **a)** Fiber flowing vertically from the extrusion nozzle and horizontally redirected by capillary D into the rotating collection container. **b)** UV-light irradiation of fiber 5 cm away from extrusion nozzle in rotating container. **c)** Collection of fiber in rotating 2 liter container. **d)** collection of fiber in rotating 4 liter container.

Fiber posttreatment: The polymerized fiber is washed in deionized water for 5 hours, then immersed two times in 190 proof ethanol (each for 6 hours) and twice in deionized water (for 1 hour each). In the last step, the fiber is dried under atmospheric conditions. Scanning electron microscopy of the fibers is carried out with a FEI Quanta 600 ESEM.

Due to the relatively large amounts of CTAB employed during fiber formation, washing of the fibers is necessary after polymerization. We find that unwashed fibers become very sticky upon drying due to thick layers of CTAB on the surface. Washing results in fibers that can easily be separated from each other after drying. Scanning electron microscopy shows that the final washing step is crucial to preserve the uniform nanoparticle coating on the surface of the fibers (Supplementary Figure 15).

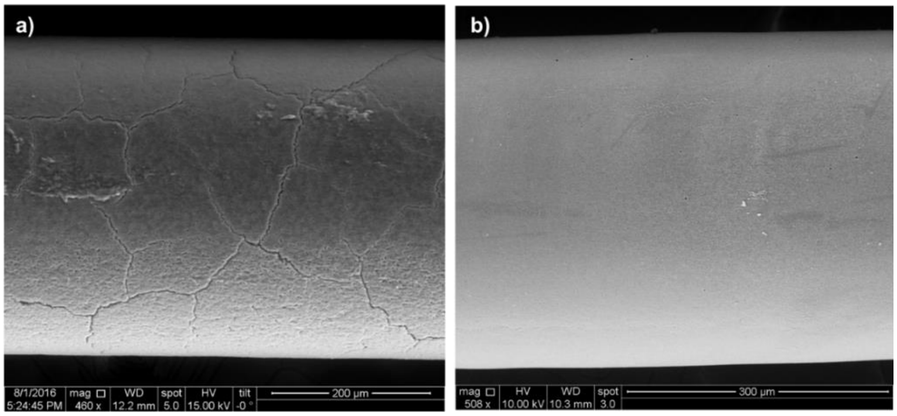

Supplementary Figure 15: *Electron micrographs demonstrating the effect of different fiber drying conditions after washing.* **a)** Fiber dried at acidic conditions. **b)** Fiber dried at alkaline condition.

The fibers dried under an acidic condition show cracks on their surface, like cracks observed in drying colloidal films. In contrast, fibers dried under alkaline conditions have crack-free surfaces with densely packed nanoparticles as shown in Supplementary Figure 11b. We also find that the cracking can be avoided by electrostatic adsorption of the polyelectrolyte poly(DADMAC) (200 – 300 kDa) in the presence of 500 mM NaCl.

Membrane flux testing. We cut three separate segments of the hollow fiber membrane to 5 cm in length and place them longitudinally and parallel on a microscope glass slide. The segments are affixed onto the glass slide with small amounts of 5-min epoxy glue near their ends. Six 1 cm long stainless steel 22-gauge metal tubes are glued at the longitudinal side of the microscope slide. A wall is formed from epoxy glue at the edge of the microscope slide that defines also divides the fibers into 3 separate chambers. On a second microscope slide the shape of this wall is reproduced and the glue is left to harden for 3 – 4 mins. The

second glass slide is then placed onto the first slide to compartmentalize the fibers into three chambers, each one completely sealed by the epoxy, but accessible through the stainless steel needles. After sufficient curing time for the glue, all the air inside the device is replaced by water. Four of the six in/outlets are closed with stoppers (stoppers are dispensing needles with cured epoxy glue inside), the feed and filtrate tubing are connected (Supplementary Figure 16). The feed liquid is stored in a Duran bottle with a dispensing screw cap and pressurized with compressed air through a regulator and a digital pressure gauge. The feed is flown into the middle chamber of the device to permeate from the outside to the inside of the fiber. For a given pressure, we measure the mass of water in a given time flowing through the membrane and calculate the flux.

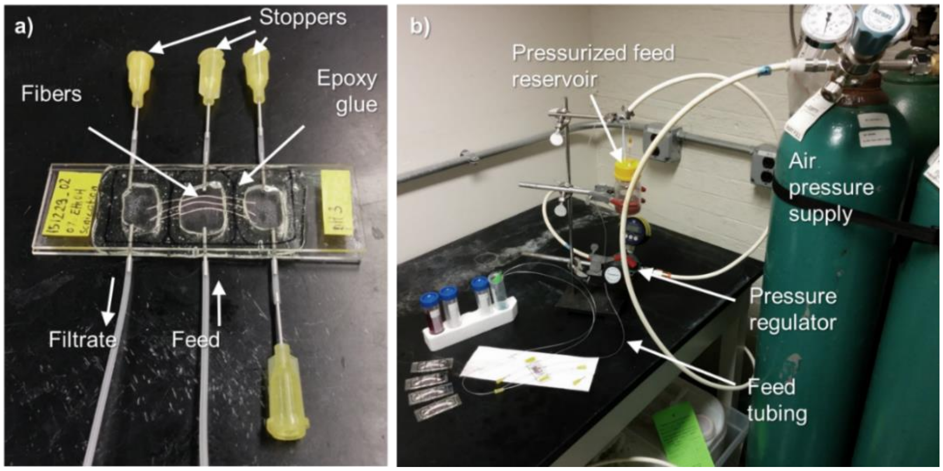

Supplementary Figure 16: *Experimental setup for flux tests*. **a)** The fibers are compartmentalized by forming an epoxy glue wall sandwiched between two microscope slides. Stainless steel needles are used as inlets and outlets. **b)** The flux test is carried out by regulating the pressure in a feed container connected to the testing device.

Supplementary References

1 Haase, M. F., Sharifi-Mood, N., Lee, D. & Stebe, K. J. In Situ Mechanical Testing of Nanostructured Bijel Fibers. *ACS nano* **10**, 6338-6344 (2016).

2 Yeh, S.-B., Chen, C.-S., Chen, W.-Y. & Huang, C.-J. Modification of silicone elastomer with zwitterionic silane for durable antifouling properties. *Langmuir* **30**, 11386-11393 (2014).

3 Frens, G. Controlled nucleation for the regulation of the particle size in monodisperse gold suspensions. *Nature* **241**, 20-22 (1973).

4 Haase, M.F., Stebe K.J. Stebe, and Lee D. Continuous Fabrication of Hierarchical and Asymmetric Bijel Microparticles, Fibers, and Membranes by Solvent Transfer-Induced Phase Separation (STRIPS). *Advanced Materials* 27, 7065-7071 (2015).

5 Herzig, E. M., White, K. A., Schofield, A. B., Poon, W. C. K., & Clegg, P. S. Bicontinuous emulsions stabilized solely by colloidal particles. arXiv preprint arXiv:0712.2213 (2007)
